# Novel open-source electronic medical records system for palliative care in low-resource settings

**DOI:** 10.1186/1472-684X-12-31

**Published:** 2013-08-14

**Authors:** Kamal G Shah, Tara Lyn Slough, Ping Teresa Yeh, Suave Gombwa, Athanase Kiromera, Z Maria Oden, Rebecca R Richards-Kortum

**Affiliations:** 1 Rice 360: Institute for Global Health Technologies, Rice University, 6100 Main St., Houston, TX 77005, USA; 2 St. Gabriel’s Hospital, Namitete, Malawi; 3 Maryland Global Initiative Corporation, Kigali, Rwanda

**Keywords:** Palliative care, Electronic medical records system, Evidence-based medicine, Africa, Database

## Abstract

**Background:**

The need for palliative care in sub-Saharan Africa is staggering: this region shoulders over 67% of the global burden of HIV/AIDS and cancer. However, provisions for these essential services remain limited and poorly integrated with national health systems in most nations. Moreover, the evidence base for palliative care in the region remains scarce. This study chronicles the development and evaluation of DataPall, an open-source electronic medical records system that can be used to track patients, manage data, and generate reports for palliative care providers in these settings.

DataPall was developed using design criteria encompassing both functional and technical objectives articulated by hospital leaders and palliative care staff at a leading palliative care center in Malawi. The database can be used with computers that run Windows XP SP 2 or newer, and does not require an internet connection for use. Subsequent to its development and implementation in two hospitals, DataPall was tested among both trained and untrained hospital staff populations on the basis of its usability with comparison to existing paper records systems as well as on the speed at which users could perform basic database functions. Additionally, all participants evaluated this program on a standard system usability scale.

**Results:**

In a study of health professionals in a Malawian hospital, DataPall enabled palliative care providers to find patients’ appointments, on average, in less than half the time required to locate the same record in current paper records. Moreover, participants generated customizable reports documenting patient records and comprehensive reports on providers’ activities with little training necessary. Participants affirmed this ease of use on the system usability scale.

**Conclusions:**

DataPall is a simple, effective electronic medical records system that can assist in developing an evidence base of clinical data for palliative care in low resource settings. The system is available at no cost, is specifically designed to chronicle care in the region, and is catered to meet the technical needs and user specifications of such facilities.

## Background

Palliative care is a rapidly emerging yet underrepresented field in sub-Saharan Africa. The need for these services has become more pronounced as a result of the HIV/AIDS epidemic, the entrenchment of tuberculosis, and the burgeoning cancer incidence and mortality. As of 2010, there were an estimated 22.9 million HIV-positive individuals in sub-Saharan Africa, representing over 67% of the global burden of the disease [1]. As of 2008, approximately 650,000 Africans were diagnosed with cancer annually, with 530,000 of these cases occurring in sub-Saharan Africa [2]. Moreover, cancer rates in Africa are expected to increase 400% by 2050 [3]. The epidemiological characteristics of this region underlie the tremendous need for comprehensive end-of-life care.

The provision of palliative care in low-resource settings like sub-Saharan Africa adopts a different structure than for most other hospital-based medical care in the region. Home-based care has long been a central tenet of end-of-life care in these settings [4]. In home-based palliative care, family or community members oversee day-to-day administration of care; common symptoms which may last an extended period of time can usually be alleviated by simple treatment. However, the need for home-based care exceeds what is currently provided in most countries [5]. Thus, care is supplemented in hospital units or by hospital support teams, who care for patients in inpatient and outpatient settings [6,7]. Patients’ continuing needs for pain management and counseling mandate repeated interaction with medical professionals across these three distinct settings. In this regard, it is challenging for providers to track patient care episodes absent a database or other patient registry system.

The growing use of EMR in the United States and Europe has been driven by the belief that these systems help improve the quality of health care. They allow for more consistent care and management from health care providers by providing access to data at the point-of-care setting. Some of the potential benefits of EMR in developing countries are preservation of clinical notes, decision support for drug ordering, program monitoring (reporting outcomes, budgets, and supplies), and long-term management of chronic diseases [8].

Numerous sources document the necessity of developing an evidence-base for palliative care in the region, yet the dearth of metrics on end-of-life care in sub-Saharan Africa severely hampers the development of such knowledge [9-12]. Despite a number of studies from Uganda that develop a preliminary evidence base for palliative care in sub-Saharan Africa, there is little research in countries in which palliative care is less integrated with the overall health system [13-15]. The African Palliative Care Association (APCA) identifies developing an evidence base for palliative care as one of four main tenets of its strategic plan. APCA aims to generate more statistics, research, and publications in order to increase this evidence base [16]. In order to develop this evidence, palliative care units must have the resources to track their own clinical data. The development of healthcare information systems in the developing world has been driven primarily by the need to report aggregate statistics to the government or funders [17]. Toward this end, this study describes the development and evaluation of DataPall, a new EMR catered to palliative care providers in low-resource settings.

## Implementation

### Sites for field assessment

DataPall was first developed for use at the Family-Centered Care Unit at St. Gabriel’s Hospital in Namitete, Malawi, and then taken to the Tiyanjane Clinic for palliative care at Queen Elizabeth Central Hospital in Blantyre. St. Gabriel’s is a private district hospital with 250 beds and is a member of the Christian Health Association of Malawi, while Queen Elizabeth Central is the largest government-run central hospital in Malawi with 1200 beds [18,19]. After one year of continued use, DataPall was updated with additional functionality at St. Gabriel’s Hospital. The authors continue to monitor these sites to assess level of satisfaction with DataPall software, improve ease of use, and troubleshoot any technical concerns. Success at the pilot sites is defined by the continued use of the software, improved organization of patient records, and a reduction in the time spent to generate reports on a unit’s activities.

### Design criteria

**Figure 1:**
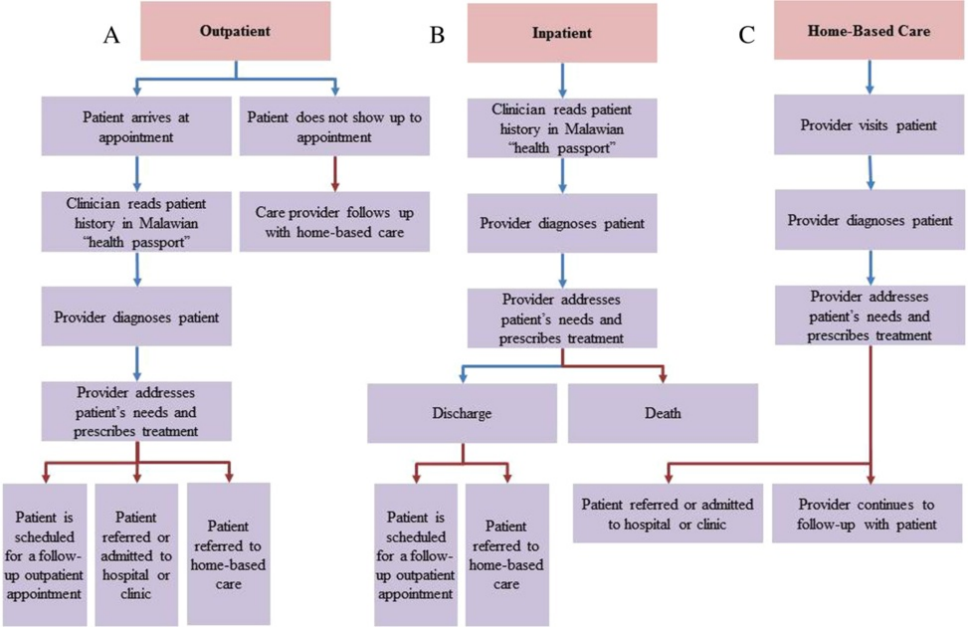
**Clinical workflow for palliative care in Malawi.** Clinical workflow includes **(A)** outpatient, **(B)** inpatient, and **(C)** home-based care settings; red arrows indicate where patient episode is recorded.

DataPall was designed in accordance with a set of functional and technical design criteria specified by physicians and palliative care providers at two field sites in Malawi, as listed in Table 1. Functionally, palliative care programs seek to monitor patients’ health status, track program development, generate patient reports, and aggregate data on the types of care provided. These tasks are more difficult as a result of variation in clinical workflow patterns across the three types of palliative care episodes: inpatient, outpatient, and home-based care (Figure 1). To achieve these design goals, the database should assign each patient a unique identification number and provide a unified interface to monitor the patient’s demographic information and appointments. Further, the database should allow for differentiation between the features of distinct patient care episodes. Finally, the database ought to be able to automatically aggregate patient data; report on patient diagnoses, outcomes, and treatments; and track services and medications provided. DataPall was developed to meet these goals while targeting a streamlined user interface, minimizing textual input, and optimizing file size.

**Table 1:** Palliative care EMR design criteria

**Database functionalities:**	**Technical specifications:**
Patient Tracking:	Open-source:
- Assign a unique identification number to each patient	- Allow for use and adoption of database with all PCs running Windows XP SP 2 or higher
- Unified interface for demographic information and appointment tracking	- Post database for download online
Program Development:	Minimize Textual Input:
- Include ability to track community health worker data, when applicable	- Use check-boxes, drop-down menus, and radio buttons whenever possible to increase input speed
- Allow for tracking of training sessions for community health workers	
Data Aggregation:	Minimize File Size:
- Compile data regarding symptoms, diagnoses, management, and treatments (all services provided) for a given time frame (monthly, quarterly, semi-annually, annually)	- Database file automatically compresses when opening and closing
Generate Report:	Streamlined User Interface:
- Allow user to generate a comprehensive report of unit’s activities during a specified time period	- Straightforward order of information input
- Design report in alignment with Malawi Ministry of Health Guidelines	- Consistency in navigation between pages
- Track medication usage	

### Use of DataPall

DataPall was developed in order to: 1) organize administrative data; 2) create a patient registry; and 3) maintain and generate reports of clinical-level data. Prior to the implementation of the DataPall EMR, recordkeeping at both field sites was conducted using paper registers provided by the Malawi Ministry of Health. These registers only offered space to record a patient’s name, age, gender, address, diagnosis, and prescribed medication. DataPall is a dual-use EMR that may be used as an administrative database (patient registry) and as a point-of-care records system meant to overcome the challenges that paper registers pose to medical recordkeeping and program evaluation.

**Figure 2:**
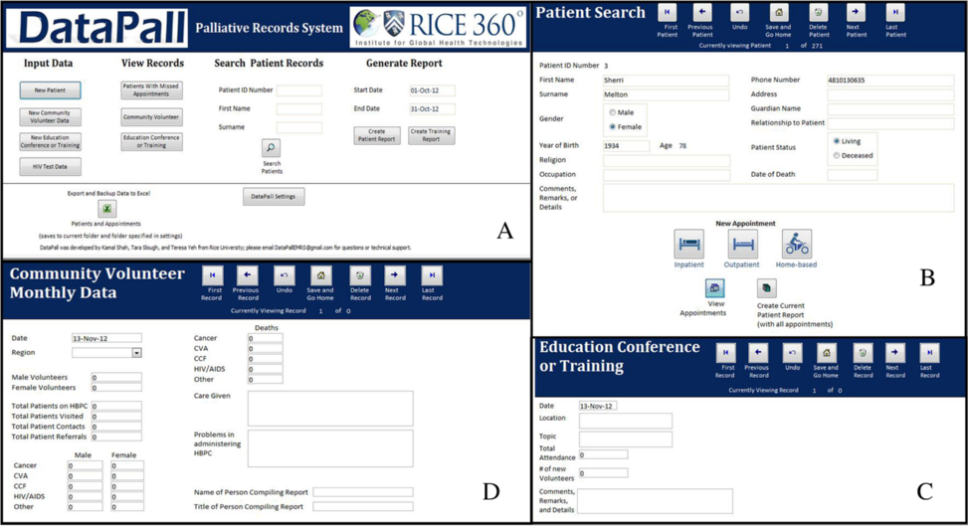
**DataPall user interface. (A)** The home screen. **(B)** The patient search screen displays patient demographic information and is used to input data. **(C)** Education conference or training data can be used to monitor palliative programs’ educational efforts. **(D)** Community volunteer data input is organized similarly to the provided paper forms.

**Figure 3:**
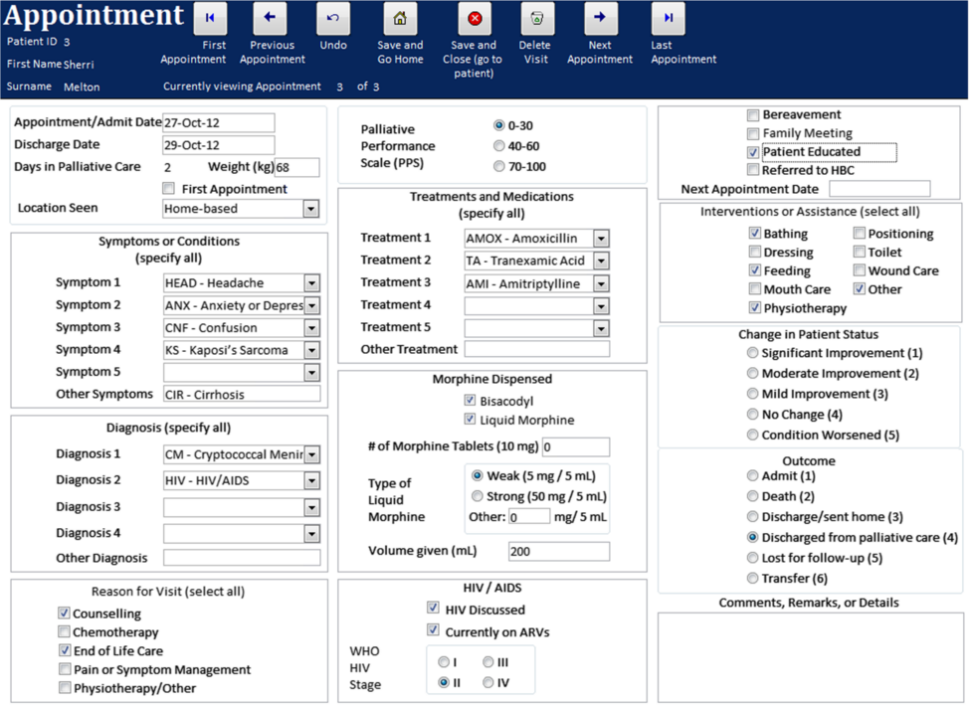
**DataPall user interface.** Patient appointment data monitors aspects of care including diagnoses, treatments, and outcome.

**Figure 4:**
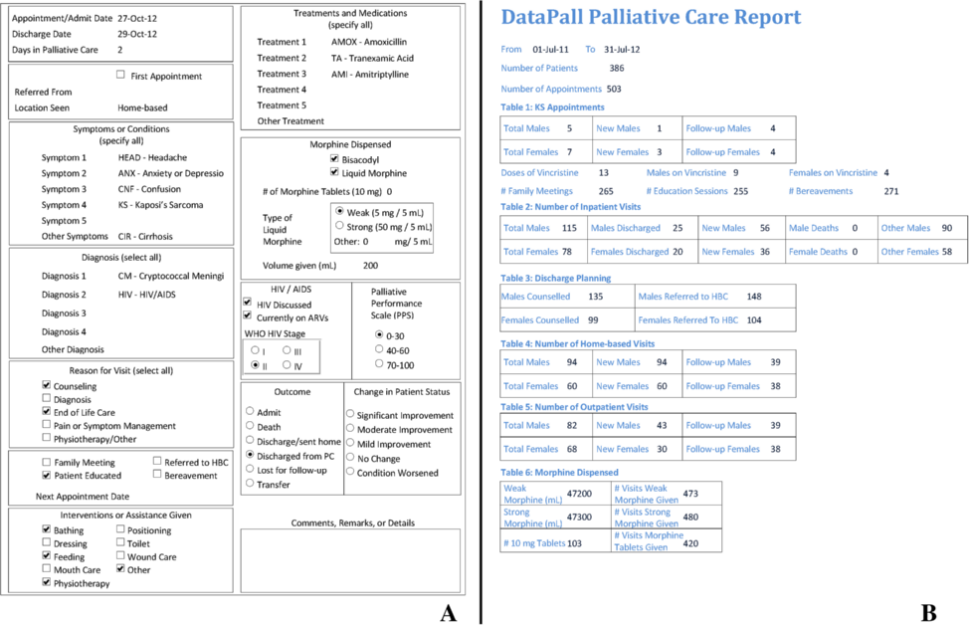
**DataPall sample reports. (A)** Individual medical history patient report has patient demographic and appointment data. **(B)** Aggregate services patient report highlights important aspects of palliative care program, including types of patients treated and the treatments rendered.

While data input to DataPall is stored in tables, the end-user does not directly interact with these tables themselves; rather, easy-to-use forms guide the user experience. A navigable interface consisting of buttons, check boxes, radio buttons, drop-down menus, datasheets, and text fields define end-user interaction (Figures 2, 3 and 4). The home screen allows for quick navigation to the various features of the database (Figure 2A). These features include: adding new patients; inputting data regarding community health workers, educational conferences or trainings; viewing records of previously entered data; searching for existing patients (Figure 2B- 2D); inputting and editing data regarding patient appointments in clinic and at home (Figure 3); and generating individual patient history reports and aggregate data for specific time periods (Figure 4).

Data for patients seen via home visits, inpatient care, or outpatient appointments are stored either as stable demographic information or as time-specific appointment information. Demographic information such as name, contact information, primary diagnosis, and HIV status is kept on a patient information page (Figure 2B), linked to a unique patient identification number which is generated subsequent to the first appointment. From the patient information page, the end-user may open an appointment information page (Figure 3) to make note of symptoms, diagnoses, treatments, outcomes, and other comments. The most common symptoms, diagnoses, treatments, and outcomes are listed for the user to choose from either a series of drop-down menus (choose more than one) or radio buttons (choose one). The user can update information on previously entered demographics or appointments at any time.

DataPall can produce several types of reports: a compilation of one individual patient’s medical history (demographic information and a snapshot of each appointment), a comprehensive eight-page aggregate patient report, and a training report. The reports that DataPall generates are automatically saved in portable document format (PDF) into the folder in which DataPall is stored. These reports show data in an organized, tabular format that can be printed, emailed, or archived.

### Infrastructure requirements

The technological design criteria were devised to maximize usability in low-resource settings like those for which DataPall is designed. First, the database (DataPall.mdb) was developed in Microsoft Access and can be run on systems with Windows XP Service Pack (SP) 2 or later. Windows XP SP 2 was readily available at the Malawian hospitals surveyed by the authors. The database requires either Microsoft Access 2007 SP 1 or the free Microsoft Access Runtime 2007 (or more recent versions of either). Virtually no configuration is necessary to run the database, which can be obtained at no cost from the authors’ institutional website, on SourceForge, or from the files supplementing this publication (Additional file 1). The database does not require an internet connection to utilize. To view the PDF reports that are generated by the database (Additional file 2), the user must have Adobe Reader (free), Foxit Reader (free), or another PDF reader that is included with most operating systems.

Within the DataPall system, textual input is minimized whenever possible, in line with the feedback provided by hospital staff members who worried about the time necessary to input appointments at slow typing rates. To achieve this specification, Microsoft Access’ button, check box, radio button, and drop-down menu options are utilized. Further, in order to maintain consistent processing speed and to avoid excessively large file sizes, the database is set to automatically compress whenever DataPall is closed. As a result, patient data can be stored in less than 10 MB in most cases (to store patient and appointment data for 9500 patients and appointments). The basic file is less than 3 MB in size without any patient data.

## Methods

### Usability tests

In order to assess the usability of DataPall in its intended setting, a study was conducted to measure the comparative advantages over extant (paper) records systems. The study involved staff members from St. Gabriel’s Hospital. Two groups of participants were recruited: ten staff members who had received 2-hour training to use the DataPall system and a sample of seven hospital staff members in other divisions of the hospital who had no training with this system, but with matched educational qualifications to the trained participants. All participants provided informed consent prior to commencing the study. Both groups of participants were given a short, two-minute tutorial on the basic functionality of the DataPall EMR prior to completing any tasks on the system. All participants were asked to complete four tasks, as follows, to compare the system to existing records systems and to evaluate the ease of the report-generating feature:

1. Participants were asked to find the most recent appointment for a sample patient (not a real patient of the hospital) using the current Malawi Ministry of Health-issued register where appointments were formerly recorded. Three appointments were noted, and patients were advised to find only the most recent appointment. This task was timed.

2. Participants were asked to find the most recent appointment for the same sample patient using the DataPall EMR system. Similarly, there were three recorded appointments for the patient, though the dates differed from the dates in the paper register. This task was timed.

3. Participants were asked to generate a comprehensive patient report in PDF format using the DataPall EMR system. This task was timed.

4. Participants were asked to use the DataPall EMR to generate an aggregate report of all the hospital’s palliative care services during a one-month span, requiring participants to set date parameters. This task was timed.

The statistical significance of observed differences in the amount of time required to locate a patient’s records in the paper register versus in the DataPall system was assessed using a Wilcoxon rank sum test. The same test was also used to assess the significance of differences in performance between trained and untrained users.

### System usability scale

In order to gauge the ease of use of the DataPall system, all participants concluded the test by completing a system usability scale comprised of ten questions [20]. Every other question was reverse-coded. The results of these surveys were aggregated by group and compared.

## Results

**Figure 5:**
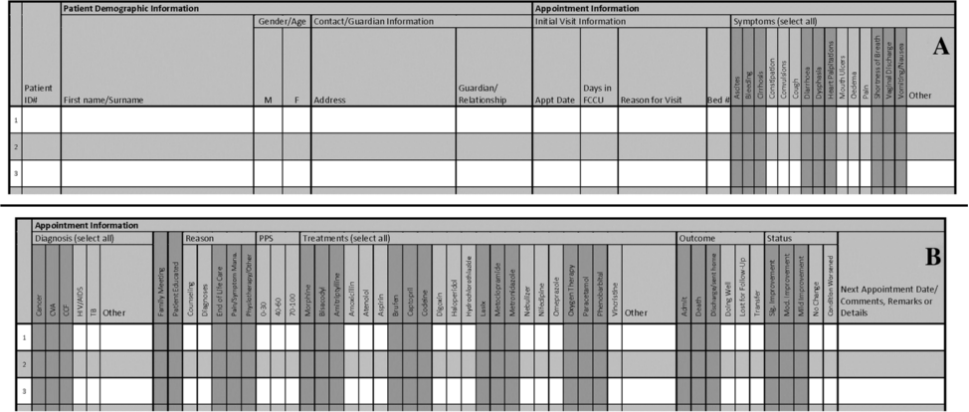
DataPall reduces the average time needed to find a recent patient’s appointment (p = 0.00057).

The goal of the DataPall EMR was to efficiently manage patient records and generate comprehensive reports on patient histories and services provided by palliative care providers. In the first two timed tasks of this study, participants were asked to locate the most recent appointment for a sample patient, first using the existing paper registers and second using the DataPall system. Figure 5 depicts the results from these two tasks. Among all participants (trained and untrained), the mean time required to locate an appointment in the paper registers was 144.9 seconds. The mean time required to locate an appointment in the DataPall system was 58.2 seconds, representing a 59.7% reduction in the time required to locate a single record. Utilizing a Wilcoxon rank sum test, the difference in performance on these two parallel tasks is significant at the p < 0.001 level. Moreover, as demonstrated in Figure 5, this relationship was observed in both trained and untrained participant groups (p < 0.05 for each). The average time needed to locate the appointment using DataPall was very similar in absolute terms between trained and untrained groups at 58.5 versus 58.0 seconds, respectively, while the median times for this task indicated a more pronounced difference between the two groups at 53. 5 seconds (trained) and 45.0 seconds (untrained). The difference in distribution between the two groups is statistically significant at the p < 0.01 level.

The DataPall EMR is designed to generate aggregate reports of clinical activity and patient history automatically, assuming that patient appointments in the given time period have been entered into the system. Using DataPall, participants generated a report of clinical data during a month-long time period in an average of 54.8 seconds. The trained users generated this report in 45.8 seconds, on average, while the untrained users required an average of 67.6 seconds to complete this task. Similarly, participants were able to generate printable patient reports for the sample patient efficiently in 42.9 seconds on average. Training did not exhibit a statistically significant difference in the amount of time required to generate these reports.

Participants in both the trained and untrained cohorts rated the usability of the DataPall system almost identically, with an overall median SUS of 77.5 with a standard deviation of 10.2. All participants rated DataPall between 65 and 97.5 on the SUS, providing relatively consistent overall consensus regarding the ease of use.

## Discussion

The results of this study indicate that DataPall provides a statistically significant improvement over paper records as a patient registry system, both among trained and untrained users. Moreover, participants’ ability to generate reports of patient history and clinical data in an average of 42.9 and 54.8 seconds, respectively, represents an even more dramatic improvement over existing records systems. Staff members indicated that generating a comprehensive report of the clinical activities over the course of a month or more using the existing paper registers could take up to 15 hours. Interestingly, although the average time needed to locate the appointment using DataPall was very similar in absolute terms between trained and untrained users, the difference in distribution between the two groups is statistically significant at the p < 0.01 level, indicating that training played a marginal but negative role on performance. We attribute this unexpected result to the increased caution with which trained users approached the task; their increased knowledge of the program caused them to take additional precautions while using it.

The mean SUS of 77.5 lies significantly above the widely accepted threshold of 70 for a passable product [21]. Moreover, there was not a statistically significant difference between the ratings of trained and untrained users, indicating that training and/or experience with the system has little or no impact on usability assessments. The SUS did present some unique challenges for some participants. While all participants spoke English, their native language, Chichewa, lacks grammatical qualifiers which limits the cultural relevancy of a Likert scale. Some participants questioned the difference between “agree” and “strongly agree” (and vice versa for disagree). However, the consistency of survey responses indicates that the confusion did not significantly affect the results of the SUS.

The absence of integration of palliative care with existing health care systems in the majority of sub-Saharan African countries impels palliative care facilities to seek funding independently from donors and granters. However, lack of reliable quantitative statistics documenting the care provided severely curtails the competitiveness of such applications. Moreover, the difficulty associated with maintaining comprehensive records of care given by palliative care providers underlies the difficulty in developing an evidence base of clinical data for palliative care in sub-Saharan Africa. Furthermore, extant paper records tend to be cumbersome to back-up. DataPall addresses these issues by allowing providers to track the care that they provide for patients and providing a straightforward way to back-up patient data to a computer and to a flash drive. The comprehensive reports generated about the activities of a unit can also assist researchers as they seek to build a stronger evidence base for palliative care in the region. These reports may also be useful to health institutions that need to provide documentation of services provided and medication used to their funders or the government.

Computer literacy is a continuing challenge for many clinics in the region. DataPall was designed with this challenge in mind. Many clinicians who were consulted for this project typed very slowly. For this reason, text-intensive fields were reduced whenever possible, instead utilizing a series of drop-down menus and check boxes. It is evident that, with practice, users can maneuver through the DataPall program with efficiency and ease. Indeed, the times for generating reports as assessed by the study are somewhat deceiving in that they assume that all back records have been input into the system. While this process takes time (a user may take up to a minute to input a new patient’s demographic and contact information and up to three for an appointment), the information that is recorded is similar to the information already recorded in registers. If used in a point-of- contact setting, the amount of extra time required to input appointment records would be marginal based on the authors’ own experience in inputting records. Thus, the DataPall EMR offers the functionality of quickly generating patient and comprehensive reports without necessitating the investment of significant time. The time saved by using DataPall to generate reports is significant.

Of the current EMR systems tailored to palliative care settings, DataPall is the first designed specifically for low-resource settings. The fields refer specifically to the information most relevant to palliative care as it is practiced in sub-Saharan Africa, most notably incorporating home-based care. Moreover, once installed, DataPall does not rely on internet connectivity to operate or maintain the database to maintain full functionality in settings where reliable high-speed internet is a rarity. Finally, DataPall is the first open-source EMR that focuses specifically on palliative care available to clinics in all locations. Through computerizing palliative care records in these settings, DataPall allows for accurate reporting and aids in computerizing medical records in resource-limited contexts.

While DataPall is catered specifically to palliative care providers in sub-Saharan Africa, it relies upon a number of core informatics principles to enhance the user experience. It provides for expanded data standardization in comparison to current records systems by providing a default set of diseases, diagnoses, symptoms, and treatments from which the user can choose whilst providing the flexibility to add, edit, and remove these parameters that are relevant to the user. Other fields, such as the core fields tracked by the Malawian Ministry of Health that are reported on the clinical activity report, remain constant. To provide additional functionality, the database can export data to a Microsoft Excel spreadsheet (“.xls” file) to enable backup or transfer of patient and appointment data to other software programs, and is set to automatically backup the database weekly. DataPall can be secured within the Microsoft Access user interface by setting a password, which encrypts the database to prevent unauthorized users from accessing patient records by prompting the user for a password when the file is opened.

**Figure 6:**
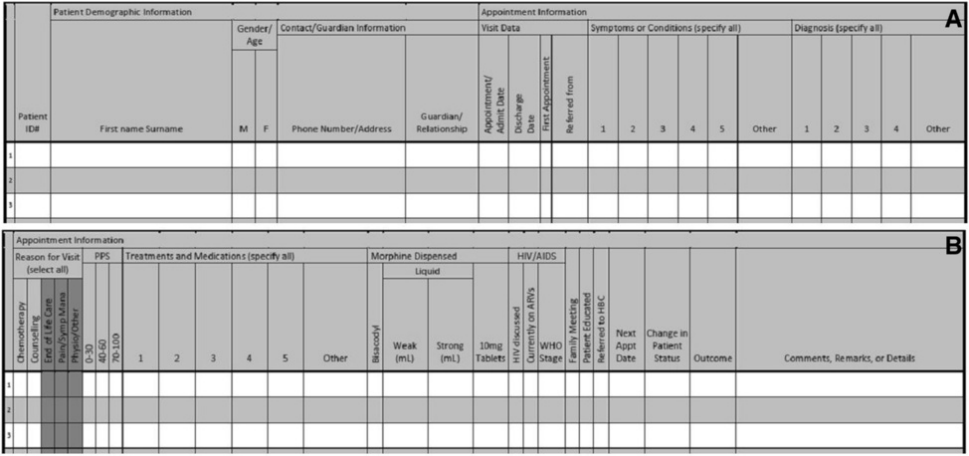
**Sample pages from the patient register compatible with DataPall and approved by the Malawi Ministry of Health.** One patient’s appointment/visit data would be input in one line across two pages of the register. **(A)** The patient’s demographic information, symptoms, and diagnoses would be recorded in the left page, and **(B)** his/her detailed appointment information would appear on the right page. The patient register, adaptable for inpatient, outpatient, or home-based care, allows for twenty unique patient appointments to be recorded over two pages.

DataPall was successfully used by field sites at St. Gabriel’s Hospital and Queen Elizabeth Central Hospital to manage palliative patient records. The sites use DataPall to supplement paper records that are used in the point-of-care context. A new system of paper records was developed and optimized for use with the DataPall EMR (Figure 6). These records consist of general patient registers for use in inpatient, outpatient, and home-based care settings and more detailed inpatient appointment sheets. The new registers, approved for use by the Malawi Ministry of Health, closely mirror the format used to input electronic records into DataPall in order to maximize efficiency of this transfer of information, and minimize the amount of text recorded.

The developers of DataPall continue to monitor the pilot sites to address ongoing concerns including: ensuring periodic backups of data; soliciting feedback on the efficiency and regularity of data input; and troubleshooting any errors in the system.

## Conclusion

DataPall is an open-source EMR that tracks patient encounters, manages data, and generates reports for palliative care providers in low-resource settings. The main benefits of DataPall include the ability to quickly view patients’ past appointments and efficiently generate comprehensive reports on all activities performed by palliative care units. The Malawian health professionals included in this study consistently evaluated DataPall as easy and efficient to use.

DataPall allows resource-constrained units to accurately quantify the services rendered in palliative care for health ministries, donors, and external organizations. Adopting the DataPall system enables providers to generate comprehensive reports on their activities, which could help build a more substantive evidence for palliative care in sub-Saharan Africa and improve patient care.

## Availability and requirements

**Project Name:** DataPall Electronic Medical Records System

**Project Page:**https://sourceforge.net/projects/datapall/

**Operating System:** Microsoft Windows XP SP 2 or higher

**Other Requirements:** Microsoft Access Runtime 2007 or Microsoft Office Access 2007 SP 1 (or newer)

**License:** GNU GPL v3

No restrictions for use by researchers, academics, or medical professionals

## Abbreviations

APCA: African Palliative Care Association; EMR: Electronic medical records system; SUS: System usability scale.

## Competing interests

This system is open source and available for free on the internet; authors expect no financial compensation for their contributions to this project. The authors declare that they have no competing interests.

## Authors’ contributions

All co-authors have made substantive intellectual contributions to the paper as follows: KGS, TLS, and PTY designed and implemented the DataPall software; created and carried out the study of its effectiveness; analyzed the data; and drafted the manuscript. AK and SG provided substantive input on the design and functionality of the DataPall software and revised the manuscript. RRK and MO oversaw the work of the team creating DataPall; advised the design of the study; and provided critical insights and revisions to the manuscript. RRK had full access to all of the data in the study and takes responsibility for the integrity of the data and the accuracy of the data analysis. All authors read and approved the final manuscript.

## Pre-publication history

The pre-publication history for this paper can be accessed here:

http://www.biomedcentral.com/1472-684X/12/31/prepub

## Supplementary Material

Additional file 1 A generic copy of the DataPall EMR. Click here for file

Additional file 2 Sample DataPall Palliative Care Report output by the DataPall EMR. Click here for file
