# Supplementary material for: Novel open-source electronic medical records system for palliative care in low-resource settings
Source: BMC Palliat Care. 2013 Aug 14;12:31. doi: 10.1186/1472-684X-12-31 (PMC3751569; doi:10.1186/1472-684X-12-31)
Supplement: Additional file 2 — Sample DataPall Palliative Care Report output by the DataPall EMR. [file 1472-684X-12-31-S2.pdf]

DataPall Palliative Care Report

From 01-Jul-12 To 31-Jul-12

Number of Patients 61

Number of Appointments 66

Table 1: KS Appointments

Total Males	2	New Males	0	Follow-up Males	2
Total Females	1	New Females	0	Follow-up Females	1

Doses of Vincristine	1	Males on Vincristine	1	Females on Vincristine	0
# Family Meetings	36	# Education Sessions	38	# Bereavements	36

Table 2: Number of Inpatient Visits

Total Males	19	Males Discharged	6	New Males	11	Male Deaths	0	Other Males	13
Total Females	13	Females Discharged	4	New Females	5	Female Deaths	0	Other Females	9

Table 3: Discharge Planning

Males Counselled	13	Males Referred to HBC	21
Females Counselled	10	Females Referred To HBC	13

Table 4: Number of Home-based Visits

Total Males	12	New Males	12	Follow-up Males	2
Total Females	9	New Females	9	Follow-up Females	2

Table 5: Number of Outpatient Visits

Total Males	8	New Males	6	Follow-up Males	2
Total Females	5	New Females	3	Follow-up Females	2

Table 6: Morphine Dispensed

Weak Morphine (mL)	7500	# Visits Weak Morphine Given	62
Strong Morphine (mL)	8200	# Visits Strong Morphine Given	63
# 10 mg Tablets	16	# Visits Morphine Tablets Given	52

Table 7: Summary of Inpatient Diagnoses

Diagnosis	Males	Females	TOTAL
AFP - Acute Flaccid Paralysis	2	0	2
BM - Bacterial Meningitis	2	1	3
CABR - Breast Cancer	1	0	1
CACX - Cervical Cancer	0	1	1
CALU - Lung Cancer	0	1	1
CALV - Liver Cancer	3	1	4
CAOE - Oesophageal Cancer	1	2	3
CAOT - Cancer (Other)	1	0	1
CAOV - Ovarian Cancer	0	1	1
CAPN - Pancreatic Cancer	3	4	7
CAST - Stomach Cancer	2	0	2
CCF - Congestive Cardiac Failure	1	1	2
CM - Cryptococcal Meningitis	1	1	2
CVA - Cerebrovascular Accident	1	0	1
HIV - HIV/AIDS	2	0	2
HPL - Hemiplegia (left side)	0	1	1
HPR - Hemiplegia (right side)	1	1	2
HPT- Hepatoma	0	1	1
HTN - Hypertension	0	2	2
LC - Liver Cirrhosis	3	1	4
NEUR - Neurological	2	0	2
PP - Paraplegia	1	2	3
QP - Quadriplegia	3	1	4
RHD - Rheumatic Heart Disease	1	0	1
SCD - Sickle Cell Disease	0	1	1
TB - (Pulmonary) Tuberculosis	1	0	1
TSS - Tropical Splenomegaly Syndrome	0	1	1
URTI - Upper Respiratory Tract Infection	1	0	1

Table 8: Summary of Outpatient Diagnoses

Diagnosis	Males	Females	TOTAL
BM - Bacterial Meningitis	1	1	2
CABR - Breast Cancer	0	1	1
CALV - Liver Cancer	0	1	1
CAOE - Oesophageal Cancer	1	0	1
CAOV - Ovarian Cancer	1	0	1
CAPR - Prostate Cancer	1	0	1
CAST - Stomach Cancer	1	1	2
CCF - Congestive Cardiac Failure	2	0	2
CVA - Cerebrovascular Accident	0	1	1
HIV - HIV/AIDS	1	1	2
HPL - Hemiplegia (left side)	0	1	1
HTN - Hypertension	1	0	1
KS - Kaposi’s Sarcoma	1	1	2
LC - Liver Cirrhosis	1	0	1
PP - Paraplegia	0	1	1
QP - Quadriplegia	0	1	1
RHD - Rheumatic Heart Disease	1	0	1
TSS - Tropical Splenomegaly Syndrome	1	0	1

Table 9: Summary of Home-based Diagnoses

Diagnosis	Males	Females	TOTAL
AFP - Acute Flaccid Paralysis	4	0	4
BM - Bacterial Meningitis	1	0	1
CALU - Lung Cancer	1	1	2
CAOE - Oesophageal Cancer	1	0	1
CAOV - Ovarian Cancer	1	1	2
CAPN - Pancreatic Cancer	1	1	2
CAPR - Prostate Cancer	3	1	4
CAST - Stomach Cancer	0	1	1
CCF - Congestive Cardiac Failure	1	0	1
CM - Cryptococcal Meningitis	1	1	2
CVA - Cerebrovascular Accident	2	1	3
HPL - Hemiplegia (left side)	0	1	1
HTN - Hypertension	1	0	1
NEUR - Neurological	1	1	2
PP - Paraplegia	0	2	2
QP - Quadriplegia	1	1	2
RHD - Rheumatic Heart Disease	1	0	1
SCD - Sickle Cell Disease	3	0	3
TB - (Pulmonary) Tuberculosis	0	2	2
TSS - Tropical Splenomegaly Syndrome	0	1	1

Table 10: Summary of Aggregate Patient Diagnoses

Diagnosis	Males	Females	TOTAL
AFP - Acute Flaccid Paralysis	6		
BM - Bacterial Meningitis	5	3	8
CABR - Breast Cancer	1	1	2
CACX - Cervical Cancer		1	
CALU - Lung Cancer	1	1	2
CALV - Liver Cancer	3	1	4
CAOE - Oesophageal Cancer	3	2	5
CAOT - Cancer (Other)	1		
CAOV - Ovarian Cancer	2	1	3
CAPN - Pancreatic Cancer	2	6	8
CAPR - Prostate Cancer	2	1	3
CAST - Stomach Cancer	5	3	8
CCF - Congestive Cardiac Failure	2	1	3
CM - Cryptococcal Meningitis	1	3	4
CVA - Cerebrovascular Accident	3	1	4
HIV - HIV/AIDS	3	1	4
HPL - Hemiplegia (left side)		3	
HPR - Hemiplegia (right side)	1	1	2
HPT- Hepatoma		1	
HTN - Hypertension	2	2	4
KS - Kaposi's Sarcoma	1	1	2
LC - Liver Cirrhosis	4	1	5
NEUR - Neurological	3	1	4
PP - Paraplegia	1	5	6
QP - Quadriplegia	2	2	4
RHD - Rheumatic Heart Disease	3		
SCD - Sickle Cell Disease	3	1	4
TB - (Pulmonary) Tuberculosis	1	2	3
TSS - Tropical Splenomegaly Syndrome	1	2	3
URTI - Upper Respiratory Tract Infection	1		

Table 11: Number of Patients by Age Group and Gender

Age (years)	Male	Female	Total
0-5:	0	0	0
6-10:	0	0	0
11-15:	0	0	0
16-45:	13	8	21
46+:	25	15	40
Total:	38	23	61

Table 12: Number of Patients Needing Assistance

Assistance	Number
Bathing:	33
Dressing:	33
Feeding:	33
Mouth Care:	41
Physiotherapy:	28
Positioning:	30
Toilet:	31
Wound Care:	33
Other:	31
Unreported:	0
Total:	61

Table 13: Change in Patient Status and Outlook

Improvement	Number
Significant:	0
Moderate:	0
Mild:	0
No Change:	0
Worsened:	0
Unreported:	61
Total:	61

Table 14: Reasons for Coming to Palliative Care

Reason	# of Patients
Counselling:	23
Diagnosis:	0
End of Life Care:	33
Pain or Symptom Management:	0
Other/ Physiotherapy:	39
Unknown/ Unreported:	0

Table 15: Palliative Performance Scale (PPS)

PPS (%)	# of Patients
0-30:	25
40-60:	41
70-100:	0
Unknown:	0

Table 16: HIV Test Results

	REACTIVE			NON-REACTIVE		
Age (years)	Male	Female	Total	Male	Female	Total
0-25	2	1	3	1	5	6
26-35	2	0	2	1	2	3
36-45	0	1	1	1	4	5
46+	0	2	2	5	7	12
Total	4	4	8	8	18	26

TOTAL Tested: 34

Table 17: Summary of Treatments

Treatment	Number of Times Given
AMI - Amitriptylline	3
AMOX - Amoxicillin	13
ASA - Aspirin	6
ATEN - Atenolol	6
BEN- Benzathine	8
BRU - Brufen	3
CAP - Captopril	3
COD - Codeine	5
CYC - Cyclizine	6
DCL - Diclofenac	5
DGX - Digoxin	6
DXM - Dexamethazole	6
FUR - Furosemide	4
GLY - Glycerin	10
HCT - Hydrochlorothiazide	4
HLP - Haloperidol	7
LAS - Lasix	9
MAG - Magnesium Salicylate	6
MCP - Metoclopramide	9
MTR - Metronidazole	9
NEB - Nebulizer	2
NFP - Nifedipine	9
O2 - Oxygen Therapy	11
OMP - Omeprazole	8
PB - Phenobarbital	8
PCM - Paracetamol	10
PIR - Piriton	6
PRO - Promethazine	3
SNK - Senakot	6
TA - Tranexamic Acid	2
TMX - Tamoxifen	3
VIN - Vincristine	7
